# To Fix or Not to Fix: Effect of Gastropexy on Esophageal Manometric Values Post Laparoscopic Sleeve Gastrectomy

**DOI:** 10.1007/s11695-026-08739-6

**Published:** 2026-05-20

**Authors:** Arsany Talaat Saber Wassef, Moamen Ali Abd El Kaream Ali, Amr Mohammed Abd El Fattah Ayad, Amir K.Abosayed, Ahmed Yahia Abd EL Dayem, Mohamed B. Hashem, Salma Omran, Ahmed Adel Shalaby Alattar

**Affiliations:** Faculty of Medicine, Cairo University Hospitals, Cairo, Egypt

**Keywords:** Gastropexy, Laparoscopic sleeve gastrectomy, Esophageal manometry, GERD, Obesity surgery

## Abstract

**Background:**

Laparoscopic sleeve gastrectomy (LSG) is widely performed for severe obesity but is associated with postoperative gastroesophageal reflux disease (GERD) and esophageal motility disorders. Gastropexy has been proposed to preserve gastric anatomy and reduce complications.

**Aim:**

To evaluate the effect of gastropexy on esophageal manometric values following LSG.

**Methods:**

A randomized controlled study was conducted on 40 patients undergoing LSG at our Hospital, (20 with gastropexy, 20 without). Esophageal manometry was performed preoperatively and 6 months postoperatively. Postoperative symptoms, complications, and percentage of total body weight loss (%TBWL) were assessed.

**Results:**

In the non-gastropexy group, there was a significant decrease in lower esophageal sphincter (LES) resting pressure and distal contractile integral (DCI) postoperatively (*p* = 0.002 and *p* = 0.01, respectively). The gastropexy group showed preserved LES pressure and DCI. Postoperative GERD symptoms, vomiting, and food intolerance were significantly less in the gastropexy group (*p* < 0.05). There was no significant difference in %TBWL between groups (*p* = 0.86). Operative time was longer in the gastropexy group (*p* < 0.001).

**Conclusion:**

Gastropexy step during LSG may play a role in preservation of the esophageal manometric values and reduces postoperative GERD and vomiting, although it increases operative time. Larger long-term trials are needed for confirmation.

## Key Points

LSG has become the most performed bariatric procedure worldwide.

Gastrointestinal symptoms (GERD, vomiting and food intolerance) are among the most challenging sequelae after LSG.

Gastropexy step may play a role in preservation of the postoperative esophageal manometric values after LSG.

Gastropexy has no negative impact on weight loss outcomes.

## Introduction

Obesity has become a global epidemic and is considered one of the leading preventable causes of morbidity and mortality worldwide. It is strongly associated with comorbidities such as type 2 diabetes, hypertension, sleep apnea, dyslipidemia, cardiovascular disease, and certain cancers [1]. Bariatric surgery is the most effective treatment for severe obesity, producing sustained weight loss and improvement in obesity-related comorbidities [2].

Laparoscopic sleeve gastrectomy (LSG) has rapidly gained popularity and has become one of the most frequently performed bariatric procedures globally due to its relative simplicity, effectiveness, and lack of intestinal bypass [3]. LSG involves resection of the gastric fundus, resulting in a tubular stomach with reduced capacity. Despite its advantages, LSG has been increasingly associated with postoperative gastroesophageal reflux disease (GERD), dysphagia, and esophageal motility disorders [4]. These complications are believed to result from increased intragastric pressure, loss of gastric compliance, disruption of the angle of His, and impairment of the lower esophageal sphincter (LES) [5].

High-resolution manometry (HRM) is a key diagnostic tool for assessing esophageal motility disorders. It provides detailed evaluation of LES pressure, distal contractile integral (DCI), and esophageal body motility. Studies have demonstrated that LSG can lead to a decrease in LES pressure and DCI, predisposing to GERD and dysphagia [5].

To mitigate these adverse effects, adjunctive techniques such as gastropexy have been proposed. Gastropexy involves fixing the sleeved stomach to surrounding structures, thereby stabilizing its position, reducing gastric twist, and preserving the anatomical angle of His. Previous studies suggest that gastropexy may reduce postoperative GERD, vomiting, and food intolerance by maintaining the integrity of the gastroesophageal junction [6].

This study aims to investigate the role of gastropexy during LSG in preserving esophagealmanometric values and reducing gastrointestinal complications. We hypothesize that gastropexy may improve functional outcomes following LSG.

## Patients and Methods

### Study Design and Setting

This study was designed as a prospective randomized controlled trial (RCT) conducted at the Department of General Surgery at, our Hospital, between May 2023 and December 2024. The study was approved by the institutional ethics committee, and written informed consent was obtained from all participants.

### Sample Size and Randomization

A total of 40 patients with severe obesity were enrolled, with sample size calculated to detect clinically significant differences in esophageal manometric outcomes, Using power and sample size calculator for intervention study; with 0.05 alpha error and power of the study, enrolment ratio of 1, 0.80, CI of 95% sample size calculated to compare Gastropexy effect on Esophageal Manometric values post laparoscopic sleeve gastrectomy is 40 patients including 10% increase to cover follow up period.(20 in each group).

Patients were randomly allocated into two equal groups (*n* = 20 each) using a concealed randomization method: Group A: Laparoscopic sleeve gastrectomy (LSG) with gastropexy and Group B: LSG without gastropexy.

### Inclusion Criteria

- Adults with BMI ≥ 40 kg/m², or ≥ 35 kg/m² with obesity-related comorbidities.
- Fit for general anesthesia.
- Failed ≥ 6 months of supervised conservative management (diet, exercise, medications).

### Exclusion Criteria

- Unfit for anesthesia.
- Previous bariatric surgery.
- Preoperative history or endoscopic evidence of GERD, esophagitis, or hiatal hernia.
- Cognitive or psychiatric impairment.

### Preoperative Assessment

#### All Patients Underwent

- Detailed history and clinical examination, focusing on obesity-related comorbidities (hypertension, diabetes, hyperlipidemia, sleep apnea).
- Laboratory tests: CBC, liver and renal function, thyroid function, fasting glucose, HbA1c, lipid profile.
- Imaging: Abdominal ultrasound to assess liver span and gallstones.
- Upper GI endoscopy: To exclude hiatal hernia or reflux esophagitis.
- High-resolution esophageal manometry (HRM): Baseline LES pressure, distal contractile integral (DCI), integrated relaxation pressure (IRP), and intra-abdominal LES length were measured following standardized protocols [1, 2].

### Surgical Technique

Patients of both groups underwent the preoperative preparation and had the surgery performed under general anesthesia in a supine position with both legs apart. The surgery was performed as standardized via 5-port technique after induction of pneumoperitonium using Veress needle through Palmer’s point. The greater curvature vascularity division was carried out using an advanced bipolar sealing device (LigaSure), beginning at about 5 cm from the pylorus and proceeding proximal with dividing gastro splenic and phreno-esophageal ligament with complete mobilization of the fundus and the angle of his. A 36-Fr calibrated bougie tube was inserted trans-orally and positioned strictly against the lesser curve, and then the sleeve line was stapled using a linear stapler. We used Endo GIA stapler from COVEDIEN for all patients, reinforcement of the stable line using oversewing technique with invagination of the proximal part of the stable line with PDS 3 − 0 continuous suture till the mid part then full thickness suturing of the rest of the staple line then secure the suture by a ligaclip. Methylene blue test was performed, and the excised stomach was removed.

**Fig. 1:**
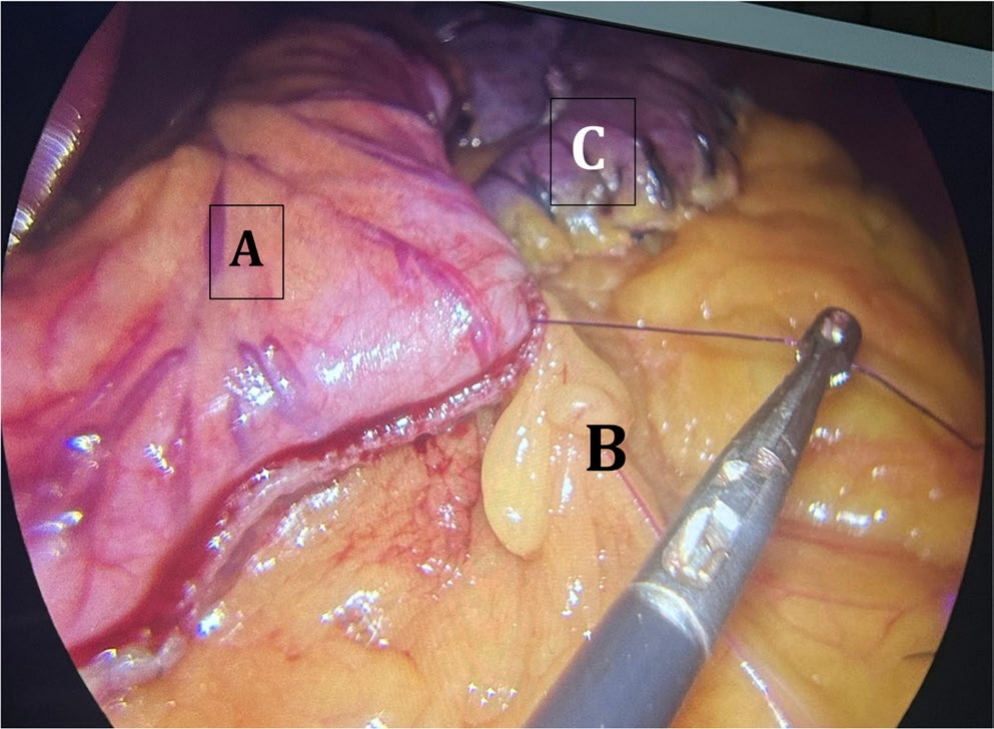
Fixation of the sleeved stomach to the pre-pancreatic fascia (Gastropexy step). **A**: Sleeved stomach **B**: Pre-pancreatic fascia **C**: Resected stomach

For patients in the gastropexy group, reinforcement of the stable line using oversewing technique with invagination of the proximal part of the stable line with PDS 3 − 0 continuous suture till the mid part then full thickness through and through suturing till the level of the incisura angularis line where pre-pancreatic fascia is sutured to the staple line using the same PDS 3 − 0 sutures in a continuous fashion 2 fixing points then full thickness suturing of the rest of the staple line then secure the suture by a ligaclip (Fig. 1).

- In Group A, gastropexy was performed by fixing the gastric sleeve to the pre-pancreatic fascia using 3 − 0 PDS sutures 2 fixing points (continuous technique).
- In Group B, no fixation was performed.

### Postoperative Care and Follow-up

1. Early ambulation and fluid intake were encouraged within 2 h.
2. Prophylactic anticoagulation and proton pump inhibitors were prescribed for 2 weeks and 3 months, respectively.
3. Patients were followed up at 6 months postoperatively, with repeat HRM to reassess esophageal motility.

Outcome measures included:

1. Primary endpoint: Changes in esophageal manometric values (LES pressure, LES length, DCI).
2. Secondary endpoints: GERD symptoms, food intolerance, vomiting, complications, operative time, hospital stay, and %TBWL.

GERD symptoms were assessed using Gastroesophageal Reflux Disease Health-Related Quality of Life (GERD-HRQL).

Patients rate each of the following statements based on their experience over the past week using a specific scale.

Scoring Scale:

- **0**: No symptom.
- **1**: Symptoms noticeable but not bothersome.
- **2**: Symptoms noticeable and bothersome but not every day.
- **3**: Symptoms bothersome every day.
- **4**: Symptoms affect daily activity.
- **5**: Symptoms are incapacitating (unable to do daily activities).

Questionnaire Items:

**Table:** 

Item	Question Description	Patient Score (0–5)
1	How bad is the heartburn?	
2	Heartburn when lying down?	
3	Heartburn when standing up?	
4	Heartburn after meals?	
5	Does heartburn change your diet?	
6	Does heartburn wake you from sleep?	
7	Do you have difficulty swallowing?	
8	Do you have pain with swallowing?	
9	Do you have bloating or a gassy feeling?	
10	If you take medication, does this affect your daily life?	

### Statistical Analysis

Data were analyzed using SPSS v21. Continuous variables were expressed as mean ± SD and compared using Student’s t test for intergroup comparison or paired t test for intragroup comparison. Categorical variables were expressed as percentages and compared using chi-square or Fisher’s exact test. Statistical significance was set at *p* ≤ 0.05.

## Results

Baseline demographic characteristics including age, sex distribution, weight, and BMI were comparable between the two study groups. The mean age was 33.9 ± 7 years in the gastropexy group versus 32.3 ± 5.9 years in the non-gastropexy group (*p* = 0.43). Similarly, sex distribution (M/F: 2/18 vs. 3/17), mean body weight (121.5 ± 11 vs. 117.2 ± 8.1 kg), and BMI (43.8 ± 5.8 vs. 43.3 ± 4.0 kg/m²) showed no statistically significant differences (*p* > 0.05).

The prevalence of comorbidities such as hypertension, diabetes mellitus, hyperlipidemia, osteoarthritis, and sleep apnea did not differ significantly between the groups (*p* > 0.05) and this is shown in Table 1.

**Table 1:** comparison of baseline data between studied groups

Variable	LSG + Gastropexy (*n* = 20)	LSG without Gastropexy (*n* = 20)	*p*-value
Age (years)	33.9 ± 7	32.3 ± 5.9	0.43
Sex (M/F)	2/18	3/17	0.63
Weight (kg)	121.5 ± 11	117.2 ± 8.1	0.16
BMI (kg/m²)	43.8 ± 5.8	43.3 ± 4.0	0.75
Comorbidity			
Hypertension	7 (35%)	7 (35%)	1.0
Diabetes Mellitus	4 (20%)	5 (25%)	0.1
Hyperlipidemia	20 (100%)	20 (100%)	1.0
Osteoarthritis	8 (40%)	7 (35%)	0.7
GERD	0 (0%)	0 (0%)	1.0
Sleep Apnea	6 (30%)	8 (40%)	0.5

Mean operative time was significantly longer in the gastropexy group (93 ± 7.36 min) compared with the non-gastropexy group (72.75 ± 4.98 min, *p* < 0.001). Postoperative morbidity showed clear advantages for gastropexy. Food intolerance occurred in 5% of gastropexy patients versus 30% of non-gastropexy patients (*p* = 0.03). Vomiting was also significantly reduced (10% vs. 40%, *p* = 0.02). GERD symptoms developed de novo in 5% versus 30%, respectively (*p* = 0.03). Rates of port site infection, bleeding and leakage were low and did not differ significantly. Mean hospital stay was nearly identical between groups (2.01 ± 1.02 vs. 2.1 ± 0.99 days, *p* = 0.7), Table 2.

**Table 2:** Comparison of intraoperative data, postoperative data and complication between studied groups

Variable		LSG + Gastropexy (*n* = 20)	LSG without Gastropexy (*n* = 20)	*p*-value
Operative Time (min) (Mean ± SD)		93 ± 7.36	72.75 ± 4.9	< 0.001
Complication	Food intolerance	1 (5%)	6 (30%)	0.03
	Port site infection	2 (10%)	1 (5%)	0.5
	Vomiting	2 (10%)	8 (40%)	0.02
	Bleeding	0 (0%)	1 (5%)	0.2
	GERD symptoms	1 (5%)	6 (30%)	0.03
	Leakage	0 (0%)	0 (0%)	1.0
Hospital Stay (days) (Mean ± SD)		2.01 ± 1.02	2.1 ± 0.99	0.7

For Esophageal Manometric Findings (Table 3) Pre- and postoperative HRM showed striking differences. In the non-gastropexy group, LES resting pressure decreased significantly (18.27 ± 3.94 to 14.62 ± 3.28 mmHg, *p* = 0.002), and DCI declined significantly (2467.75 ± 594.68 to 2012.50 ± 523.0 mmHg·s·cm, *p* = 0.01). By contrast, in the gastropexy group, LES pressure and DCI were preserved (no significant change, *p* > 0.05). LES intra-abdominal length decreased slightly in both groups, but without statistical significance. Median LES (IRP4) increased slightly in both groups but without statistical significant.

**Table 3:** Comparison of esophageal manometric findings between studied groups

Parameter	Group	Pre-op (Mean ± SD)	Post-op (Mean ± SD)	*p*-value
LES resting pressure	Gastropexy	16.69 ± 4.67	15.68 ± 4.21	0.47
	Non-gastropexy	18.27 ± 3.94	14.62 ± 3.28	0.002
LES intra-abdominal length (cm)	Gastropexy	2.72 ± 0.59	2.62 ± 0.61	0.6
	Non-gastropexy	2.32 ± 0.53	2.16 ± 0.52	0.34
DCI	Gastropexy	2185.35 ± 708.87	2314.75 ± 773.62	0.58
	Non-gastropexy	2467.75 ± 594.68	2012.50 ± 523.00	0.01
Median LES (IRP4)	Gastropexy	4.66 ± 2.38	5.14 ± 3.62	0.453
	Non-gastropexy	4.73 ± 2.45	5.19 ± 3.56	0.449

At 6 months, mean %TBWL was 23.11 ± 1.60% in the gastropexy group versus 23.03 ± 1.37% in the non-gastropexy group (*p* = 0.86), (Table 4)

**Table 4:** Comparison of total body weight loss (TBWL) % between studied groups

Group	TBWL% (6 months, Mean ± SD)	*p*-value
LSG + Gastropexy	23.11 ± 1.60	0.86
LSG without Gastropexy	23.03 ± 1.37	

## Discussion

Laparoscopic sleeve gastrectomy (LSG) has become one of the most common bariatric operations and is associated with higher rates of GERD compared to other procedures. Obesity itself is a strong risk factor for GERD, and LSG may exacerbate reflux due to anatomical changes and increased intragastric pressure [7, 8].

Gastropexy has been proposed to stabilize the stomach after LSG by fixing it to the diaphragm or peripancreatic fascia, which may prevent gastric torsion, reduce postoperative nausea, and improve esophageal manometric values. Studies have suggested that fixation reduces gastric twist, functional stenosis, and postoperative dysphagia [9, 10].

Fixation to the pre-pancreatic fascia considered as strong and stable anchoring structure not affected by weight loss as the greater omentum in omentopexy and that may help in stabilization of the sleeve and prevent upward migration hence may play a role in preservation of the esophageal manometric values.

AbdEllatif et al. [11] reported using gastropexy successfully after failure of endoscopic dilatation. Kizilkaya et al. [12] demonstrated that posterior fixation with fibrin sealant reduced postoperative dysphagia. Other prospective studies confirmed that gastric fixation reduces the incidence of twist, nausea, and vomiting compared with classic LSG [13].

Our findings agree with Flølo et al. [14], Abou-Ashour [6], and Afaneh et al. [15], who found no demographic differences between groups, and demonstrated that gastropexy (or omentopexy) reduced postoperative GI symptoms. Similarly, Okasha and Soliman [16] found comparable comorbidities but reduced morbidities with gastropexy.

Operative time was significantly longer in gastropexy cases, consistent with Abou-Ashour [6], Abosayed and Mostafa [17], Labib [18], and Okasha and Soliman [16]. This difference is attributed to the extra fixation step, but was not associated with prolonged hospital stay. Contrastingly, Elbalshy et al. [19] reported no significant difference in operative time.

Our study showed no difference in hospital stay, consistent with Zarzycki et al. [20], although Okasha and Soliman [16] found longer stay in gastropexy. Thus, gastropexy did not increase hospitalization duration, indicating that the additional surgical step did not negatively impact early recovery.

Postoperative bleeding was rare, similar to Abosayed [17], Al Haddad [21]and Labib [18]. Gastropexy significantly reduced vomiting, food intolerance, and GERD symptoms compared to standard LSG.

The increase in GERD symptoms after LSG is related to dissection of the angle of His, loss of sling fibers, high intragastric pressure, and impaired LES function [15]Our results confirm lower incidence of GERD with gastropexy, which may contribute to preserve the anti-reflux mechanism.GERD symptoms were assessed using Gastroesophageal Reflux Disease Health-Related Quality of Life (GERD-HRQL) .There was no hiatal hernia pre-operatively (exclusion criteria) and post-operatively in the studied groups.

At 6 months, LES pressure and DCI were preserved in the gastropexy group, while they decreased significantly in the non-gastropexy group. While there is no significant difference between the two groups regarding the intra-abdominal LES length and median LES IRP4s with normal esophageal motility pattern pre and post in both group, Our results are in line with Mohamed Ramadan et al. [22]who reported significant decreases in LES pressure and DCI after LSG without gastropexy.

these observed Manometric differences may be indirectly related to the gastropexy step.

6 months is a relatively short period of follow up, longer duration with a bigger sample size is recommended to assess the Durability of the LES esophageal values and GERD symptoms with adding upper GI endoscopy and 24 h ph-metry for objective analysis of GERD symptoms.

In our study, there was no significant difference between the studied groups regarding mean %TBWL was 23.11 ± 1.60% in the gastropexy group versus 23.03 ± 1.37% in the non-gastropexy group (*p* = 0.86).

In supporting with Abosayed [17] and Abdullah [23] found that there was no significant differences between the gastropexy and non gastropexy group regarding mean TBWL %.

This demonstrates that gastropexy has no negative impact on weight loss outcomes, indicating that its functional benefits do not compromise the metabolic efficacy of LSG.

### Strengths and Limitations

Strengths of this study include is one of a few prospective studies assessing the impact of gastropexy on esophageal Manometric values Post Laparoscopic Sleeve Gastrectomy in the literature, its randomized controlled design and the use of HRM which provides objective and reproducible assessment of the esophageal motility and LES function.

Limitations include the single-center design, relatively small sample size, and limited follow-up duration, which restrict long-term generalizability. Commenting on LES relaxing pressure, LES intra-abdominal length, DCI, IRP4 and motility patterns only considered a simplified methodology as a full standardized HRM interpretation and analysis of the data according Chicago Classification framework would greatly enhance the study.

## Conclusion

Gastropexy performed during LSG may play a role in preservation of the esophageal manometric values and decreases postoperative GERD, vomiting, and food intolerance, despite increasing operative time. It may be considered as an adjunct step in bariatric surgery to improve functional outcomes.

## Data Availability

No datasets were generated or analysed during the current study.
